# A Dual-Mode Memristor-Based Oscillator for Energy-Efficient Biomedical Wireless Systems

**DOI:** 10.3390/mi17040393

**Published:** 2026-03-24

**Authors:** Imen Barraj, Mohamed Masmoudi

**Affiliations:** 1 Department of Computer Engineering, College of Computer Engineering and Sciences, Prince Sattam Bin Abdulaziz University, Al-Kharj 11942, Saudi Arabia; 2 Systems Integration & Emerging Energies (SI2E), Electrical Engineering Department, National Engineering School of Sfax, University of Sfax, Sfax 3038, Tunisia

**Keywords:** memristor emulator, DTMOS, dual-mode oscillator, ring VCO, chirp generator, biomedical signal conditioning, wireless telemetry

## Abstract

This paper presents a novel dual-mode memristor-based ring oscillator designed for energy-efficient, wireless biomedical signal conditioning systems. The proposed architecture leverages a compact DTMOS memristor emulator, consisting of only two transistors and one capacitor, to replace the conventional NMOS pull-down devices in a three-stage PMOS ring oscillator. This integration enables two distinct operating modes within a single compact core: a fixed-frequency mode for stable clock generation and carrier synthesis, and a programmable chirp mode for frequency-modulated signal generation. The fixed-frequency mode achieves continuous tuning from 3.142 GHz to 4.017 GHz via varactor control, with an ultra-low power consumption of only 111 µW at 4.017 GHz. The chirp mode generates linear frequency sweeps starting from 0.8 GHz, with the sweep range independently controllable through the state capacitor value and the pulse width of the control signal (*SW_Chirp_*). Designed in a standard 0.18 µm CMOS process, the oscillator exhibits a low phase noise of −87.82 dBc/Hz at a 1 MHz offset for the three-stage configuration, improving to −94.3 dBc/Hz for the five-stage design. The overall frequency coverage spans 0.8–4.017 GHz, representing a 133.6% fractional range. The calculated figure of merit (FoM) is −169.45 dBc/Hz. Experimental validation using a discrete CD4007 prototype confirms the oscillation principle, while comprehensive simulations demonstrate robust performance across process corners and temperature variations. With its zero-static-power memristor core, wide tunability, and dual-mode reconfigurability, the proposed oscillator is ideally suited for multi-standard wireless biomedical applications, including implantable telemetry, neural stimulation, ultra-wideband (UWB) transmitters, and non-contact vital sign monitoring.

## 1. Introduction

The rapid proliferation of wearable and implantable medical devices is revolutionizing personalized healthcare, enabling continuous monitoring, diagnosis, and therapeutic intervention [1,2]. These systems critically depend on their analog front-end (AFE) circuits to acquire weak, noisy physiological signals, such as electrocardiogram (ECG), electroencephalogram (EEG), and electromyogram (EMG), with high fidelity under stringent constraints of power, area, and adaptability [3]. Conventional AFE designs, typically built from operational amplifiers and passive filters, are fundamentally linear and static. They lack the inherent intelligence to adapt their parameters dynamically in response to changing signal characteristics or environmental noise, often requiring complex digital post-processing or power-hungry tuning circuits to achieve acceptable performance [4]. This limitation becomes particularly acute in next-generation biomedical applications that demand not only ultra-low-power operation for extended battery life or energy-harvesting compatibility, but also advanced functionality such as adaptive filtering, programmable gain control, and on-chip signal synthesis for calibration or neural stimulation. Furthermore, recent comprehensive reviews have surveyed the rapid progress in memristors for emerging applications in neuromorphic computing and biomedical systems, highlighting both the challenges and opportunities in this field [5].

To address this need for adaptive, low-power analog processing, the field of neuromorphic engineering has drawn significant interest, seeking to mimic the efficiency and plasticity of biological neural systems [6]. A cornerstone of this approach is the memristor, a theoretically predicted nonlinear circuit element whose resistance (memristance) is modulated by the history of applied charge or flux [7]. This inherent memory and state-dependent behavior make the memristor a fundamentally adaptive component, ideal for implementing tunable filters, oscillators, and synapses in brain-inspired circuits [8]. However, the widespread integration of physical memristive devices in standard CMOS processes remains a technological challenge, primarily due to issues with variability, yield, and modeling. It is important to recognize that the memristor concept has been realized through diverse physical mechanisms beyond the original theoretical formulation. Electrical memristors based on resistive switching in nanoscale silicon structures have demonstrated the ability to support spike-timing-dependent plasticity, establishing the foundation for neuromorphic computing systems where memristors serve as high-density, high-connectivity synaptic elements [9]. Optoelectronic memristors have emerged that combine the advantages of photonics and electronics, with all-optically controlled analog memristors based on InGaZnO demonstrating memconductance reversibly tunable over continuous ranges by varying the wavelength of controlling light, enabling applications in optoelectronic spiking neural networks [10]. More recently, nanofluidic ionic memristors have been developed that exploit ion transport through nanoscale confined fluidic systems, where internal ionic conductance states depend on historical voltage, mimicking biological ion carriers and offering unique advantages for brain-inspired computing and biosensing applications at ultra-low power consumption [11]. While these physical memristive devices offer unique and powerful capabilities for specialized applications, ranging from high-density neuromorphic computing to optoelectronic processing and bio-inspired ionic systems, each presents distinct integration challenges when combined with standard CMOS processes, often requiring specialized materials, post-processing steps, or non-standard fabrication flows that complicate system-on-chip integration. As a practical and immediate alternative that circumvents these integration barriers, CMOS-based memristor emulators have emerged as a vital research pathway, allowing designers to explore novel adaptive circuit architectures using only standard transistors and capacitors available in any commercial CMOS process.

Recent research in memristor emulation has strategically pivoted toward minimalist, transistor-efficient designs to reduce static power, silicon area, and parasitic effects, attributes paramount for biomedical integration [12,13]. Our prior work introduced a Dynamic Threshold MOS (DTMOS)-based memristor emulator (DMEC), which achieves floating operation, zero static power consumption, and high-frequency performance using only two transistors and one capacitor [14]. This emulator provides a compact, power-efficient, and electronically tunable core for building adaptive analog circuits.

A critical and highly versatile building block in any AFE or communication subsystem is the oscillator. Oscillators generate the essential clocking signals for synchronous systems, provide carrier waves for wireless telemetry, and can directly synthesize stimulation waveforms for neuromodulation [15,16]. Traditionally, oscillator design involves a fixed LC tank or ring of inverters, producing a frequency determined by passive components or delay stages. Integrating a memristor into an oscillator’s feedback loop introduces a unique opportunity: the oscillation frequency, waveform, and mode can become dynamically controllable by the memristor’s state, which itself is a function of the signal history. This enables oscillators that are reconfigurable, adaptive, and capable of complex signal generation without digital overhead.

In this context, this paper presents a novel reconfigurable memristor-based oscillator designed specifically for energy-efficient, wireless biomedical signal conditioning systems. The proposed architecture features a dual-mode ring oscillator capable of operating in two distinct configurations: a fixed-frequency mode for stable clock generation, suitable for system timing and carrier wave synthesis; and a chirp generation mode for frequency-modulated signal synthesis, enabling advanced biomedical applications.

The remainder of this paper is organized as follows: Section 2 briefly reviews the operating principle of the DTMOS memristor emulator. Section 3 details the circuit design and theoretical analysis of the proposed dual-mode oscillator. Section 4 presents comprehensive simulation results, validating performance across modes, frequencies, and power conditions. Section 5 provides a comparative discussion with prior art and explores potential biomedical applications. Finally, Section 6 concludes the paper.

## 2. DTMOS Memristor Emulator

The proposed multi-mode oscillator leverages a compact floating memristor emulator as its fundamental adaptive component. This section concisely reviews the emulator’s topology and, crucially, characterizes the specific electrical properties that enable its function as a programmable control element within the oscillator. A more detailed derivation of its operating principles can be found in our prior work [14].

**Figure 1:**
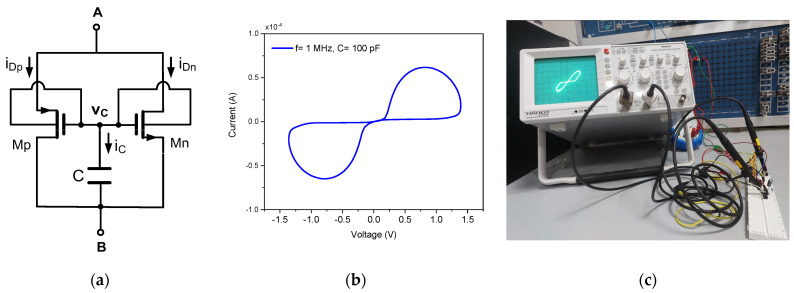
DTMOS-based memristor emulator: (**a**) circuit design; (**b**) pinched hysteresis loop at 1 MHz; and (**c**) experimental validation using a CD4007 circuit.

The emulator, depicted in Figure 1a, is designed for minimalism and full CMOS compatibility. Its core consists of only two dynamic threshold MOS transistors, one PMOS (Mp) and one NMOS (Mn), and a single state capacitor (C). In the DTMOS configuration, the gate and body terminals are connected, rendering the threshold voltage ($V_{TH}$) a dynamic function of the gate-source potential. This intrinsic property is exploited to generate a voltage-controlled, non-volatile resistance. The two transistors are interconnected in a complementary, symmetrical topology between the floating terminals A and B, forming the main current path. Their common gate node is connected to the capacitor C, which stores the internal state variable as charge, with voltage $v_{C}$. When an AC signal is applied across terminals A and B, the transistors conduct alternately. The effective channel resistance of the conducting transistor in each half-cycle is dynamically modulated by $v_{C}$, producing a pinched hysteresis loop in the current–voltage (I–V) characteristic, the signature fingerprint of memristive behavior, as illustrated in Figure 1b. The fundamental operation of this topology has been verified experimentally. Figure 1c shows the discrete prototype implemented with the CD4007 IC, and Figure 1c presents the corresponding measured PHL, confirming successful emulation.

The memristive behavior of the proposed DMEC arises from the interaction between the DTMOS transistors and the integrating capacitor. The capacitor voltage $v_{C}$ serves as the state variable, storing the history of the input signal applied across terminals A and B. For a positive input voltage $v_{AB}>0$, the NMOS transistor $M_{n}$ conducts, while the PMOS transistor $M_{p}$ remains off. The terminal current $i_{AB}$ is given by the drain current of $M_{n}$, which depends on both the instantaneous input voltage $v_{AB}$ and the state voltage $v_{C}$, the latter modulating the threshold voltage $V_{TH,n}$ through the body effect. This coupling between $v_{C}$ and $V_{TH,n}$ is the key mechanism that enables the device to exhibit memory: as charge accumulates on C, the threshold voltage shifts, altering the channel conductivity and thus the resistance between terminals A and B.

For a sinusoidal input $v_{AB}(t)=A_{m}sin(\omega t)$, the memductance $W=i_{AB}/v_{AB}$ can be expressed as [13]:

(1) $$W(t)\approx W_{0}+\Delta Wcos(\omega t)$$

where

- $W_{0}=k_{n}\left[(1-\alpha_{n})V_{C0}-V_{TH0,n}\right]$ is the constant memductance offset;
- $\Delta W=\frac{k_{n}^{2}\beta_{n}A_{m}V_{C0}(1-\alpha_{n}{)}^{2}}{C\omega }$ is the amplitude of the time-varying memductance.

$k_{n}$ is the transconductance parameter of $M_{n}$, $V_{TH0,n}$ is its zero-bias threshold voltage, $\alpha_{n}$ is the body-effect coefficient, and $\beta_{n}$ is a coupling factor related to the DTMOS device capacitance.

Equation (1) reveals several critical insights into the emulator’s operation. First, the memductance consists of a constant term $W_{0}$ and a time-varying component ΔWcos(ωt). This time-varying term is directly proportional to the input amplitude $A_{m}$ and inversely proportional to both the capacitance C and the frequency ω. Consequently, the strength of the memristive effect—the degree to which the device exhibits history-dependent resistance—can be precisely tuned by adjusting these parameters, offering designers flexible control over the emulator’s dynamic behavior. A fundamental consequence of the inverse relationship ΔW∝1/ω is the characteristic frequency-dependent hysteresis. At low frequencies, ΔW is large, producing a wide pinched hysteresis loop in the current–voltage (i–v) characteristic. As the operating frequency increases, the loop area progressively contracts. In the high-frequency limit where ω→∞, ΔW→0, and the device behaves as an ordinary linear resistor with constant conductance $W_{0}$. This behavior satisfies one of the three canonical fingerprints of a memristor: the hysteresis lobe area decreases monotonically with increasing excitation frequency, confirming that the proposed circuit faithfully emulates true memristive dynamics. The inverse relationship between ΔW and C also provides a straightforward and practical design knob. Smaller capacitors yield stronger memristive modulation, manifested as wider hysteresis loops and more pronounced nonlinearity. Conversely, larger capacitors reduce the modulation depth, pushing the device toward linear resistive behavior. This tunability via capacitance allows the same core circuit to be tailored for diverse applications by simply selecting or programming the value of C, without requiring any modification to the transistor-level design. Finally, the dependence of ΔW on the input amplitude $A_{m}$ introduces an additional dimension of programmability. Larger input signals produce stronger memristive modulation, meaning that the device’s effective impedance becomes amplitude-dependent. This property can be strategically exploited in biomedical front-end systems for functions such as automatic gain control or compressive sensing, where larger biosignal amplitudes naturally experience different effective impedances, enabling adaptive signal conditioning without the need for complex control circuitry. Together, these insights confirm that Equation (1) provides not only a mathematical description of the DMEC’s behavior but also a roadmap for its practical application in adaptive, low-power biomedical systems.

Equation (1) encapsulates the fundamental fingerprint of memristive behavior: the pinched hysteresis loop (PHL) in the current–voltage (i–v) plane, whose lobe area contracts as the input frequency ω increases. This frequency-dependent behavior confirms the dynamic memory characteristics of the proposed emulator. Furthermore, the presence of a well-defined pinch at the origin (i=0, v=0) satisfies the second memristor fingerprint, while the dependence on the history of the input (encoded in $v_{C}$) satisfies the third. Thus, Equation (1) provides a compact yet complete mathematical description of the DMEC’s memristive operation, serving as the foundation for its integration into more complex adaptive circuits.

Furthermore, an important characteristic of the proposed DTMOS memristor emulator is its volatile memconductance behavior. The state variable is stored as charge on the capacitor C, which determines the voltage $v_{C}(t)$ that modulates the threshold voltages of the DTMOS transistors according to Equation (1). In the absence of an applied signal across terminals A and B, this charge gradually leaks away through gate and junction leakage currents inherent to the MOS transistors. The retention time can be estimated from the discharge rate $dv_{C}/dt=I_{leak}/C$, where $I_{leak}$ is the total leakage current. For fixed-frequency mode, where C is in the sub-picofarad range, the retention time is on the order of microseconds; for chirp mode, where C is in the picofarad range, the retention time extends to milliseconds. Critically, during normal oscillator operation, the continuous AC signal across the memristor terminals constantly refreshes the state voltage through the bidirectional current $i_{AB}(t)$ that flows during each oscillation cycle. This self-refreshing mechanism ensures that the memductance remains stable and the oscillator maintains its programmed frequency indefinitely as long as power is applied. The volatile nature is not a limitation but rather a design feature that offers several advantages. First, the oscillator can be easily reset by briefly removing the input signal or applying a discharge pulse, enabling precise control over the chirp generation and allowing the chirp to be initiated from a known initial state. Second, the natural evolution of the state voltage in response to the integrated input current enables the linear frequency sweep in chirp mode without requiring external ramp generators or complex control circuitry. Third, the emulator integrates seamlessly into standard CMOS processes, using only conventional transistors and capacitors without requiring exotic nonvolatile storage elements, post-processing steps, or specialized materials. For our target applications, implantable telemetry, neural stimulation, and ultra-wideband transmitters, the volatile behavior is well suited, as these systems typically operate continuously or in short bursts where the self-refreshing mechanism maintains stable operation, and power-down states can be managed through simple reinitialization.

The DMEC possesses several key attributes that make it an ideal candidate for building adaptive oscillators, particularly for area- and power-constrained integrated systems:

- Floating and passive operation: As a true two-terminal device requiring no DC bias, it can be seamlessly inserted into oscillator feedback loops or frequency-setting networks without complicating biasing schemes or introducing static power paths.
- Electronic tunability: The memristance of the DMEC is electronically tunable through two complementary mechanisms, enhancing its versatility for both analog and mixed-signal systems. Primarily, the memristance is modulated by the state capacitor C, which sets the core time constant of the emulator. In an integrated implementation, C can be realized as a voltage-controlled MOS capacitor (MOSCAP), allowing smooth, continuous analog tuning of the memristance via an external control voltage. This provides a direct analog approach for adjusting frequency and waveform characteristics in oscillator applications. Additionally, the memristor state, and hence its resistance, can be programmed or dynamically adjusted by applying a train of voltage pulses across its terminals. Each pulse incrementally charges or discharges the state capacitor, shifting the memristance in a discrete yet controllable manner. This pulse-driven programmability enables digital calibration, weight updates in neuromorphic contexts, and event-based reconfiguration, making the DMEC suitable not only for purely analog adaptive circuits but also for digitally assisted and programmable oscillator designs.
- High-frequency capability: The minimalist topology minimizes parasitic effects, enabling operation up to several hundred megahertz, suitable for both biomedical signal conditioning and RF-related functions.
- Compact layout footprint: At the layout level, the emulator consists of only two DTMOS transistors. In a standard CMOS process, these can be realized in an extremely compact active area. The capacitor C can be implemented either as a discrete external component for prototyping or as an integrated MOSCAP for full on-chip integration. When implemented on-chip, the capacitor value can be made tunable via an external control voltage applied to the well terminal, enabling precise electronic adjustment of the memristor’s time constant without significant area penalty.
- Zero static power: The absence of static current paths ensures that the core emulator contributes no quiescent power overhead, aligning perfectly with energy-constrained and energy-harvesting applications.
- Minimal overhead in system integration: Due to its two-transistor core and integrable capacitor, the addition of the DMEC to a larger system-on-chip (SoC), such as a biomedical front-end or a neuromorphic processor, incurs negligible area overhead. This makes it a practical adaptive element that enhances functionality without compromising the form factor or cost of the final integrated circuit.

Collectively, these attributes, especially the state-dependent resistance, capacitive tunability, tiny layout footprint, and zero static power, make the DMEC a foundational building block for designing oscillators that are not only reconfigurable and adaptive but also highly integrable and efficient.

## 3. Proposed Oscillator Architecture

This section introduces the novel oscillator architecture designed for advanced biomedical signal conditioning. The proposed circuit is a reconfigurable dual-mode ring oscillator capable of generating both fixed-frequency and chirp signals. The design exploits the state-dependent resistance and tunability of the DMEC to achieve adaptive functionality with minimal hardware overhead.

### 3.1. Circuit Topology

**Figure 2:**
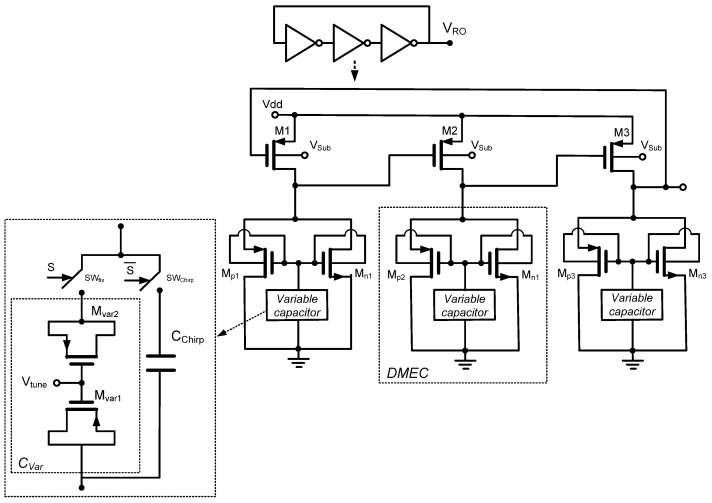
Proposed memristor-based dual-mode ring oscillator.

The architecture of the proposed dual-mode oscillator is built based on a PMOS ring oscillator whose frequency is directly controlled by the programmable load presented by DTMOS memristor emulators. This topology was selected for its inherent simplicity, low transistor count, and the direct, monotonic relationship between the memristor’s state and the oscillation period. The core of the oscillator, shown in Figure 2, is a three-stage ring oscillator where each stage is a PMOS inverter with a memristive load. A standard inverter stage consists of a PMOS pull-up transistor and an NMOS pull-down transistor. In this design, the NMOS pull-down device is replaced by the DTMOS memristor emulator, which acts as a programmable resistive load to ground. This results in a PMOS-driven, memristor-loaded inverter (MLI). The three MLI stages are connected in a ring configuration (output of stage N connected to input of stage N + 1), with the output of the third stage fed back to the input of the first stage to satisfy the Barkhausen criterion for oscillation. The primary advantage of this PMOS–memristor topology is that the switching speed of each stage, and thus the overall oscillation frequency, is dominated by the RC time constant formed by the output node capacitance C_out_ and the effective pull-down resistance, which is the memristance M(q).

### 3.2. Oscillation Principle and Frequency Control Mechanism

A programmable capacitor network, depicted in Figure 2, replaces the fixed state capacitor in the DMEC to enable explicit dual-mode operation. The network comprises two parallel branches: a voltage-controlled MOS varactor ($C_{var}$) for fixed-frequency mode and a larger fixed capacitor ($C_{chirp}$) for chirp generation. A digital mode-select signal S controls complementary switches ($SW_{fix}$, $SW_{chirp}$) that activate only one branch at a time. In fixed-frequency mode (S=0), the emulator is connected to a small-value, voltage-tuned MOS varactor ($C_{var}$) through switch $SW_{fix}$. The varactor is adjusted by an external tuning voltage $V_{tune}$, allowing fine, continuous control of the capacitance in the sub-pF range. This small capacitance ensures a short memristor time constant, forcing the state voltage to track the input rapidly and resulting in stable, fixed-frequency oscillation. The frequency can be electronically tuned via $V_{tune}$. In chirp mode (S=1), switch $SW_{chirp}$ connects the emulator to a larger, fixed capacitor ($C_{chirp}$), typically in the range of picofarad. This capacitor can be implemented either as an integrated capacitor or as an external discrete component, depending on the application’s precision and area constraints. The larger capacitance introduces a long time constant, causing the state voltage to integrate the input signal over many oscillation cycles. This produces a slow ramp in memristance and, consequently, a linear frequency sweep.

The dual-mode operation of the proposed oscillator can be rigorously described using the memristor state equations derived for the DMEC [13]. The oscillation frequency $f_{osc}$ of the three-stage ring oscillator is governed by the stage delay $\tau_{d}$, which is dominated by the discharge time through the memristor:

(2) $$f_{osc}\approx \frac{1}{2N\tau_{d}},\tau_{d}\approx R_{mem}\left(t\right)\cdot C_{L}$$

where N=3 is the number of stages, $C_{L}$ is the output node capacitance, and $R_{mem}(t)=1/W(t)$ is the instantaneous memristance given by Equation (1) from the DMEC analysis [13].

When the state capacitor C is small (<0.1 pF), the time-varying component ΔW in Equation (1) becomes negligible, as it averages to zero over each cycle due to rapid settling of the state voltage. Thus, $W(t)\approx W_{0}$, a constant, and Equation (2) simplifies to:

(3) $$f_{fixed}\approx \frac{W_{0}}{2NC_{L}}$$

The memductance offset $W_{0}$ depends on the quiescent state voltage $V_{C0}$, which is tunable via $V_{tune}$ applied to the varactor, enabling electronic frequency tuning in fixed-frequency mode.

For capacitor values in the picofarad range (>1 pF), the time constant given by Equation (4) becomes significantly larger than the oscillation period:

(4) $$\tau =\frac{2\pi C}{k_{n}\beta_{n}A_{m}(1-\alpha_{n})}$$

In this regime, the AC component of the state voltage is suppressed, and $v_{C}(t)$ evolves quasi-statically according to the integrated input charge:

(5) $$v_{C}\left(t\right)\approx V_{C0}+\frac{\beta_{n}\langle i_{AB}\rangle }{C}t$$

where $\left\langle i_{AB}\right\rangle$ is the average memristor current over a cycle. Since $v_{C}(t)$ increases linearly with time, substituting Equation (4) into the memductance expression yields a linearly ramping memductance:

(6) $$W\left(t\right)\approx W_{0}+mt,m=\frac{\beta_{n}k_{n}(1-\alpha_{n})\langle i_{AB}\rangle }{C}$$

Substituting Equation (5) into Equation (2) gives the time-varying chirp frequency:

(7) $$f_{chirp}\left(t\right)\approx \frac{W_{0}+mt}{2NC_{L}}$$

Equations (3) and (7) provide the design equations for the two operating modes: fixed-frequency oscillation when C is small, and linear frequency chirp when C is large. The transition between modes occurs when the time constant τ becomes comparable to the oscillation period, which can be controlled through the value of C.

It is important to emphasize that the linear frequency sweep described by Equation (7) arises from a quasi-static integration regime fundamentally distinct from conventional exponential charging dynamics. When the state capacitor C is sufficiently large (typically exceeding 1 pF), the time constant $\tau =2\pi C/(k_{n}\beta_{n}A_{m}(1-\alpha_{n}))$ becomes significantly larger than the oscillation period $T_{osc}$. Under this condition, the state voltage $v_{C}(t)$ does not track the instantaneous AC signal but rather integrates the average current $\left\langle i_{AB}\right\rangle$ over many oscillation cycles, yielding a linear ramp as expressed in Equation (5). The linearity of this frequency sweep can be understood by distinguishing between two fundamentally different charging regimes. In conventional passive RC networks, a capacitor charged through a fixed resistor from a constant voltage source exhibits exponential $e^{-t/RC}$ behavior. However, in our active feedback oscillator topology, the memristor emulator is embedded within a self-sustaining oscillatory loop. The state capacitor C integrates the bidirectional current $i_{AB}(t)$ that flows through the complementary DTMOS pair during each oscillation cycle. Over many cycles, the net charge accumulation is proportional to the time integral of the average current, which remains approximately constant because the oscillation amplitude and waveform shape maintain stability throughout the chirp duration. This behavior is directly analogous to an operational amplifier integrator circuit, which produces a precise linear ramp when integrating a constant current, precisely the condition achieved in our design when C is sufficiently large.

The proposed dual-mode oscillator achieves frequency control through a fundamentally different mechanism than conventional oscillator designs, offering several distinct advantages for biomedical and adaptive systems. Unlike traditional voltage-controlled oscillators, which rely on varactor tuning or current-starving techniques that often introduce nonlinearity, limited range, and high sensitivity to supply noise, our approach leverages the inherent memory and analog programmability of the memristor. This enables seamless dual-mode operation, fixed-frequency and chirp generation, within the same compact topology, eliminating the need for separate clock and waveform generation circuits. Compared to digitally controlled oscillators, which require power-hungry frequency-locked loops or lookup tables, our design provides continuous analog tuning via capacitor C with zero static power overhead. Moreover, unlike LC-based chirp generators that demand large inductors and complex modulation circuits, our design uses only two transistors and a capacitor, achieving both low- and high-frequency range chirp capability in an ultra-compact, CMOS-friendly footprint. The memristor’s state-dependent resistance not only simplifies the architecture but also introduces intrinsic adaptability, allowing the oscillator to respond dynamically to input conditions, an attribute absent in fixed linear oscillators. Consequently, this work demonstrates a scalable, low-power, and reconfigurable frequency generation strategy that is particularly suited for wireless biomedical systems where area, power, and functional versatility are critical constraints.

## 4. Simulation Results and Performance Analysis

This section presents comprehensive simulation results validating the functionality and performance of the proposed dual-mode memristor-based oscillator. All simulations were performed in a standard 0.18 µm CMOS process, with the DTMOS memristor emulator implemented using the compact two-transistor, one-capacitor topology described in Section 2.

### 4.1. Fixed-Frequency Mode

As described above, the variation in oscillation frequency is accomplished by electronically adjusting the state capacitance of the memristor emulator using inversion-mode MOS (I-MOS) varactors integrated into the DMEC core. In the proposed architecture, for the fixed operation mode, two NMOS transistors $M_{var1}$ and $M_{var2}$ are configured as I-MOS varactors, replacing the fixed state capacitor C. The drain and source terminals of both transistors are tied together to form the first terminal, which is connected to the common gate node of the DTMOS pair in the DMEC. The gate terminals of $M_{var1}$ and $M_{var2}$ are connected to form the control terminal, which is driven by the external tuning voltage $V_{tune}$. This configuration allows the effective capacitance at the state node to be varied electronically. In this circuit configuration, when the control voltage $V_{tune}$ is increased, the varactors enter deeper inversion, thereby increasing the effective capacitance at the state node. This increased capacitance slows the rate of change in the state voltage $v_{C}(t)$, which in turn increases the memristance $R_{mem}$ according to Equation (1). Since the oscillation frequency is inversely proportional to $R_{mem}$, the overall effect is a reduction in oscillation frequency. Conversely, decreasing $V_{tune}$ reduces the varactor capacitance, leading to lower memristance and higher oscillation frequency. Thus, the I-MOS varactors provide a smooth, voltage-controlled tuning mechanism for the memristor-based oscillator, enabling wide frequency programmability in the fixed-frequency mode while maintaining the compact, two-transistor core of the DMEC. Table 1 provides the sizing of the transistors used in the proposed dual oscillator.

The simulation results for the proposed dual-mode oscillator were obtained through systematic variation in several key design parameters: the supply voltage $V_{dd}$, the I-MOS varactor gate control voltage $V_{tune}$, $V_{sub}$ applied exclusively to the PMOS pull-up transistors, and the width W of the I-MOS varactor transistors.

**Figure 3:**
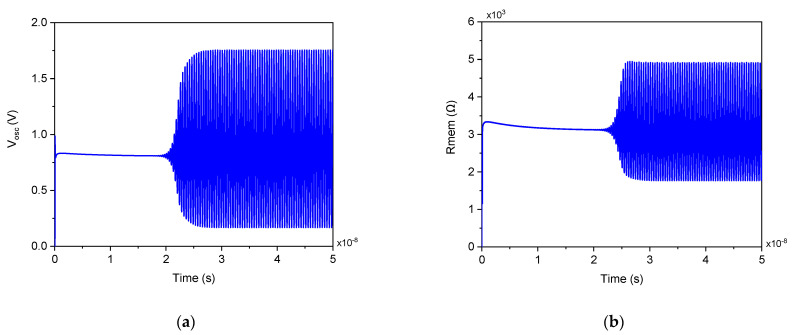
Transient simulation of the proposed dual-mode oscillator: (**a**) Output voltage waveform; (**b**) Corresponding instantaneous memristance $R_{mem}(t)$ for $V_{tune}=1$ V and varactor width $W_{var}=1\;µm$.

The transient simulation of the proposed dual-mode oscillator for $V_{tune}=1$ V and varactor width $W_{var}=1\;µm$, is depicted in Figure 3. As shown, the output voltage exhibits a stable oscillation at approximately 3.858 GHz with a peak-to-peak amplitude of 1.58 V, while the corresponding memristance $R_{mem}(t)$ oscillates synchronously between 1.9 kΩ and 4.91 kΩ given an average of 3.405 kΩ, confirming the dynamic, state-dependent resistance modulation intrinsic to the memristive feedback loop. As shown, transient analysis of the proposed oscillator reveals a fundamental memristive behavior: the instantaneous memristance $R_{mem}(t)$ oscillates synchronously with the output voltage, alternating periodically between two distinct resistance states. This phenomenon arises directly from the state-governed dynamics of the DTMOS-based emulator. During each oscillation cycle, the voltage applied across the memristor drives a bidirectional current $i_{AB}(t)$, which continuously charges and discharges the state capacitor C according to the memristor’s governing equation $C\;dv_{C}/dt=\;i_{AB}(t)$. Consequently, the state voltage $v_{C}(t)$ modulates rhythmically, and because the memristance is inversely proportional to $v_{C}(t)$, as expressed in Equation (1), $R_{mem}(t)$ likewise oscillates. This cyclic resistance variation is not an artifact but a signature of memristive operation; it embodies the pinched hysteresis characteristic in the time domain, confirming that the emulator behaves as a true floating memristor rather than a static linear resistor. The observed symmetry and regularity of the resistance swing further attest to the balanced push-pull action of the complementary DTMOS pair and the proper integration of the memristor into the oscillator’s feedback loop. This dynamic memristance modulation is essential for enabling the circuit’s dual-mode functionality, whether stabilizing frequency in fixed mode or generating chirps through controlled state evolution, and demonstrates the design’s fidelity to memristive system theory. Although the instantaneous memristance $R_{mem}(t)$ oscillates between two discrete values due to the alternating conduction of the complementary DTMOS pair, the oscillation frequency remains stable because the time-averaged memristance $\left\langle R_{mem}\right\rangle$ is constant over each full cycle. This balance results from the symmetric push–pull operation of the emulator, where the higher and lower resistance states contribute equally to the total propagation delay, yielding a fixed output frequency despite the underlying memristance modulation.

The PMOS varactors are biased such that their source/drain terminals are connected to the state node at DC voltage $V_{C0}$V. The tuning voltage $V_{tune}$ is applied to the gate terminal, ranging from 0.4 V to 1.8 V. This ensures that the effective gate–source voltage $V_{gs}=V_{tune}-V_{C0}$ sweeps from negative to positive values, moving the varactor from accumulation through depletion into inversion, thereby providing the necessary capacitance variation for frequency tuning. At low tuning voltages ($V_{tune}<0.4$ V), the PMOS varactor operates in deep accumulation, where the capacitance characteristic becomes non-ideal due to parasitic gate overlap and leakage effects. This results in a deviation from the expected monotonic frequency tuning trend, yielding slightly lower oscillation frequencies than predicted by the ideal model. For practical applications, the usable tuning range is therefore defined for $V_{tune}\geq 0.4$ V, where the varactor exhibits well-behaved inversion/accumulation transitions.

**Figure 4:**
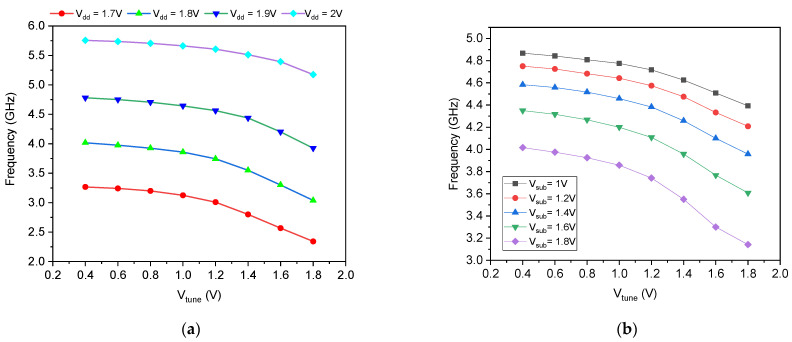
Variation in frequency for different values of (**a**) *V_dd_* and (**b**) *V_sub_*.

The oscillation frequency of the proposed dual-mode oscillator in fixed-frequency configuration was characterized first across a range of supply voltages ($V_{DD}$) and substrate bias voltages ($V_{sub}$) applied exclusively to the PMOS pull-up transistors $M_{P1}$, $M_{P2}$, and $M_{P3}$ of the three-stage ring oscillator core. The PMOS varactor width was fixed at $w_{var}=1\;\mu m$, and the tuning voltage $V_{tune}\;$was swept from 0.4 V to 1.8 V. The complete frequency data set is summarized in Table 2, with Figure 4a illustrating the $V_{DD}$ dependence and Figure 4b depicting the $V_{sub}$ dependence.

Figure 4b presents the oscillation frequency as a function of $V_{tune}$ for five different substrate bias voltages applied to the well terminals of $M_{P1-3}$, with $V_{sub}$ ranging from 1.0 V to 1.8 V. In contrast to the $V_{DD}$ trend, increasing $V_{sub}$ monotonically decreases the oscillation frequency. At $V_{tune}=0.4$ V, the frequency drops from 4.867 GHz at $V_{sub}=1.0$ V to 4.017 GHz at $V_{sub}=1.8$ V, a 17.5% reduction. This inverse relationship is a direct consequence of the body effect in PMOS transistors. As $V_{sub}$ rises (moving closer to or above the source voltage $V_{DD}$), the source–body voltage $V_{SB}$ increases, which elevates the threshold voltage $V_{TH}$ of the PMOS devices. A higher $V_{TH}$ reduces the overdrive voltage for a given $V_{GS}$, weakening the pull-up current and increasing the rise time delay. The effect is consistent across the entire $V_{tune}$ range, with the highest frequencies always observed at the lowest $V_{sub}$. Importantly, because $V_{sub}$ is applied only to the oscillator core transistors and not to the memristor emulator itself, this tuning mechanism operates independently of the memristor’s state, providing an orthogonal degree of frequency control without perturbing the memristive dynamics.

As shown in Figure 4a, the oscillation frequency exhibits a strong positive correlation with $V_{DD}$. At a fixed $V_{tune}=0.4$ V, increasing the supply voltage from 1.7 V to 2.0 V raises the frequency from 3.267 GHz to 5.756 GHz, a 76% improvement. This behavior is attributed to the increased overdrive voltage ($V_{GS}-V_{TH}$) of the PMOS pull-up transistors, which enhances their current-drive capability and reduces the output node rise time. Since the oscillation frequency is inversely proportional to the propagation delay per stage, any reduction in delay directly translates to higher $f_{osc}$. Notably, the frequency sensitivity to $V_{DD}$ is more pronounced at lower $V_{tune}$ values, where the memristance is minimal and the stage delay is dominated by the PMOS drive strength. At higher $V_{tune}$, the memristance increases and becomes the limiting factor, compressing the frequency spread across $V_{DD}$. This behavior confirms that both the memristor and the PMOS pull-up devices contribute to the overall delay, offering two independent knobs for frequency control. The power consumption of the proposed dual-mode oscillator was characterized across supply voltage $V_{DD}$ and tuning voltage $V_{tune}$ variations. For a fixed $V_{DD}$, the power consumption remained virtually independent of $V_{tune}$, with measured values of 78.3 µW at $V_{DD}=1.7$ V, 111 µW at $V_{DD}=1.8$ V, 149 µW at $V_{DD}=1.9$ V, and 192 µW at $V_{DD}=2.0$ V, regardless of the specific $V_{tune}$ setting. This behavior confirms that the DMEC consumes zero static power, as its operation is purely passive and driven solely by the oscillating signal.

Furthermore, the total power consumption in our oscillator arises from three fundamental mechanisms: dynamic switching losses, short-circuit current, and any static bias currents. The dynamic switching power is dissipated primarily in the PMOS pull-up transistors as they charge and discharge the output node capacitances during each oscillation cycle. This component is inherent to any switching circuit and scales with frequency, capacitance, and supply voltage. The short-circuit power occurs during the brief interval when both the PMOS pull-up and the memristor path conduct simultaneously during switching transitions, creating a temporary direct path from supply to ground. Critically, the memristor emulator itself contributes negligibly to the total power dissipation. This is because the DTMOS-based memristor is a passive two-terminal element with no DC bias path. Unlike a conventional NMOS pull-down transistor, which requires a static connection to ground and conducts continuous subthreshold leakage, the memristor emulator conducts current only during switching events and does so symmetrically, charging the state capacitor during one half-cycle and discharging it during the next. The absence of any DC path to ground or supply also eliminates the leakage currents that typically plague conventional CMOS oscillators, particularly at elevated temperatures. The conventional oscillator dissipates significantly more power due to three factors: the NMOS pull-down transistors contribute both dynamic switching losses and static leakage, the short-circuit current is substantially larger because of the abrupt switching characteristics of complementary CMOS inverters, and the presence of direct DC paths through the NMOS devices allows continuous subthreshold conduction. In contrast, our memristor-based architecture replaces the lossy NMOS pull-down with a lossless reactive element, fundamentally altering the power dissipation profile. The memristor emulator consumes no DC power and contributes only reactive energy transfer to the system. The total power consumption is dominated by the unavoidable dynamic switching of the PMOS transistors and the reduced short-circuit current, both of which are minimized through the memristor’s gradual switching characteristics.

**Figure 5:**
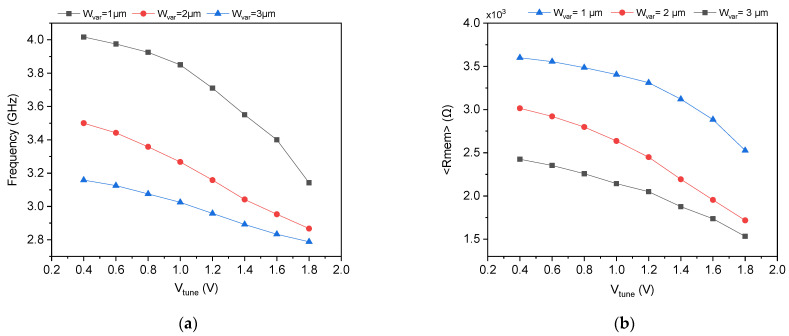
Simulation results of the three-stage dual-mode oscillator: (**a**) Oscillation frequency and (**b**) Corresponding average memristance for different $W_{var}$.

**Figure 6:**
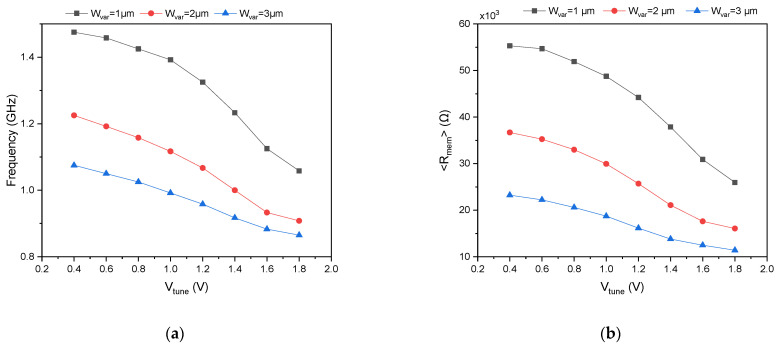
Simulation results of the five-stage dual-mode oscillator: (**a**) oscillation frequency and (**b**) corresponding average memristance for different $W_{var}$.

To further investigate the tunability of the proposed dual-mode oscillator, the oscillation frequency and average memristance $\left\langle R_{mem}\right\rangle$ were characterized as functions of the varactor tuning voltage $V_{tune}$ for three different PMOS varactor widths: $W_{var}=1$, 2, and 3 μm, and two ring oscillator stage configurations: three-stage and five-stage. The supply voltage and substrate bias were fixed at $V_{DD}=1.8$ V and $V_{sub}=1.8$ V for all simulations. The complete dataset is summarized in Table 3 for the three-stage topology and Table 4 for the five stage topology, with graphical representations provided in Figure 5 and Figure 6, respectively. As shown in Figure 5a and Table 3, increasing the varactor width $W_{var}$ reduces the oscillation frequency across the entire $V_{tune}$ range. For a fixed $V_{tune}=0.4$ V, the frequency decreases from 4.017 GHz at $W_{var}=1$ μm to 3.158 GHz at $W_{var}=3$ μm, a 21.4% reduction. This inverse relationship is explained by the proportional dependence of varactor capacitance on gate area: $C_{var}\propto W_{varac}\cdot L$. A wider varactor presents a larger effective capacitance at the memristor’s state node, which increases the time constant and slows the evolution of the state voltage $V_{c}\left(t\right).\;$Consequently, the memristance $R_{mem}(t)$ remains higher on average, as confirmed by Figure 5b, where $\left\langle R_{mem}\right\rangle$ at $V_{tune}=0.4$ V decreases from 3.6 kΩ at $W_{var}=1$ μm to 2.242 kΩ at $W_{varac}=3$ μm. Since the oscillation frequency is inversely proportional to $R_{mem}$, this increase in memristance directly suppresses $f_{osc}$. Notably, the frequency tuning range (defined as $f_{max}/f_{min}$ over $V_{tune}=0.4$–1.8 V) expands with increasing varactor width. For $W_{varac}=1$ μm, the tuning ratio is 1.28× (4.017 GHz/3.142 GHz), while for $W_{var}=3$ μm, it widens to 1.32× (3.158 GHz/2.808 GHz). This improvement is attributed to the enhanced capacitance modulation depth of larger varactors, which experience a greater absolute change in capacitance as $V_{tune}$ sweeps from accumulation to inversion. The wider capacitance swing produces a larger variation in $v_{C}(t)$ and thus a broader memristance range, translating into an extended frequency tuning span. However, this comes at the cost of reduced maximum frequency, as the larger minimum capacitance of wide varactors limits how low $R_{mem}$ can go even at minimal $V_{tune}$.

Figure 6 and Table 4 present the corresponding results for the five-stage ring oscillator configuration. As expected, increasing the number of stages from three to five reduces the oscillation frequency by approximately 2.7 to 3.8 times across all conditions, consistent with the fundamental relationship $f_{osc}\propto 1/N$. For example, at $W_{var}=1$ μm and $V_{tune}=0.4$ V, the five-stage oscillator operates at 1.475 GHz compared to 4.017 GHz for the three-stage design. This frequency scaling enables the same core topology to cover different application bands without redesigning the delay cells: three stages for GHz-range wireless telemetry and five stages for sub-GHz baseband processing or intermediate-frequency signal conditioning. Furthermore, the strong correlation between $\left\langle R_{mem}\right\rangle$ and oscillation frequency across all configurations confirms that the memristor is the dominant frequency-setting element in this design. The ability to predict and control $f_{osc}$ through $\left\langle R_{mem}\right\rangle$, whether by varying $V_{tune}$, $W_{var}$, or N, validates the theoretical model developed in Section 3 and establishes the DMEC as a reliable, repeatable, and integrable adaptive component for next-generation biomedical microsystems.

The spectral purity of the proposed dual-mode oscillator was evaluated through phase noise simulations for both three-stage and five-stage configurations at their respective center frequencies. For the three-stage oscillator operating at 4.017 GHz, the phase noise at a 1 MHz offset was measured as −87.82 dBc/Hz. For the five-stage oscillator configuration, the phase noise was measured at a center frequency of 1.475 GHz. At 1 MHz offset, the phase noise improved to −94.3 dBc/Hz, representing a 6.5 dB enhancement compared to the three-stage design. This improvement is attributed to two factors: first, the lower oscillation frequency inherently reduces phase noise for a given technology; second, the increased number of stages distributes the delay more evenly, reducing the effective jitter contribution per stage.

**Figure 7:**
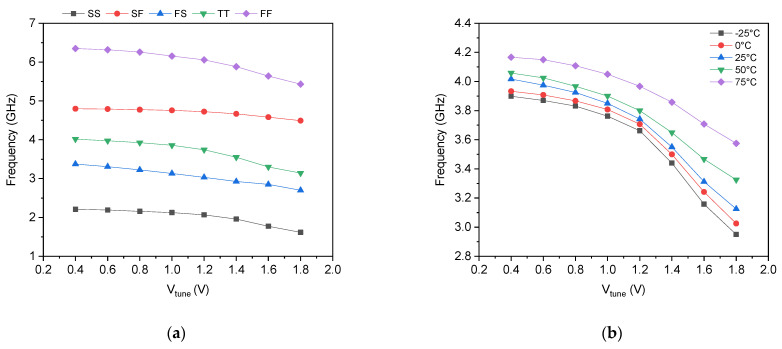
Oscillation frequency versus tuning voltage $V_{tune}$ at different (**a**) Process corners and (**b**) temperatures, simulated at $V_{DD}=1.8$ V, $V_{sub}=1.8$ V, and $W_{var}=1$ µm.

The robustness of the proposed dual-mode oscillator was evaluated through process corner simulations across the five standard CMOS corners: TT (typical–typical), FF (fast–fast), SS (slow–slow), FS (fast–slow), and SF (slow–fast). Simulations were performed at $V_{DD}=1.8$ V, $V_{sub}=1.8$ V, and $W_{var}=1$ µm, with $V_{tune}$ swept from 0.4 V to 1.8 V. As depicted in Figure 7a, the oscillation frequency exhibits the expected dependence on process conditions: the FF corner yields the highest frequencies, ranging from 6.35 GHz at $V_{tune}=0.4$ V to 5.433 GHz at $V_{tune}=1.8$ V, while the SS corner produces the lowest frequencies, from 2.208 GHz to 1.617 GHz over the same tuning range. This significant spread, approximately 2.9 times between FF and SS at $V_{tune}=0.4$ V, is attributed to the combined effects of faster carrier mobility, lower threshold voltages, and reduced memristance in fast corners, all of which decrease stage delay and elevate oscillation frequency. The SF and FS corners exhibit intermediate frequency responses, with SF consistently higher than FS due to the asymmetric impact of NMOS versus PMOS strength on the memristor-loaded PMOS inverter topology. Importantly, the oscillator maintained stable, continuous oscillation across all corners and tuning voltages, and the memristor’s characteristic pinched hysteresis loop remained clearly observable in each case. These results confirm that the proposed architecture is inherently robust to global process variations and can be reliably manufactured across different process splits. The thermal stability of the proposed dual-mode oscillator was characterized additionally across −25 °C to 75 °C at $V_{DD}=1.8$ V, $V_{sub}=1.8$ V, and $W_{var}=1$ µm. As depicted in Figure 7b, the oscillation frequency exhibits a positive temperature coefficient across all $V_{tune}$ values, increasing from 3.899 GHz at −25 °C to 4.167 GHz at 75 °C for $V_{tune}=0.4$ V. This variation arises because the threshold voltage reduction in the DTMOS devices outweighs mobility degradation with rising temperature, decreasing memristance and stage delay. For biomedical applications, this inherent stability ensures reliable operation across diverse environmental conditions, from cold storage to body temperature and febrile states, while eliminating the need for external temperature sensors or calibration loops. The monotonic frequency–temperature relationship also offers potential for dual-purpose use as an on-chip temperature sensor, further enhancing the oscillator’s utility in resource-constrained implantable and wearable systems. For biomedical applications requiring tight frequency tolerance, the observed corner-dependent frequency shifts can be readily compensated through the oscillator’s multiple tuning approaches, enabling post-fabrication calibration without additional circuitry or area overhead. Our proposed oscillator architecture offers four independent tuning mechanisms, each with distinct characteristics suitable for different calibration stages. The state capacitor C serves as the most powerful tuning knob, as it directly controls the memristor’s time constant and can shift the oscillation frequency over an order of magnitude. The supply voltage $V_{DD}$ provides a wide tuning range of ±43% around the center frequency, making it ideal for coarse corner correction, though with linear power scaling. The varactor control voltage $V_{tune}$ offers ±28% continuous fine-tuning with zero static power overhead, as it only sets the voltage on MOS varactor gates with negligible DC leakage. Additionally, the substrate bias $V_{sub}$ provides a ±17% tuning range that can be dedicated to temperature compensation without perturbing the primary controls.

**Figure 8:**
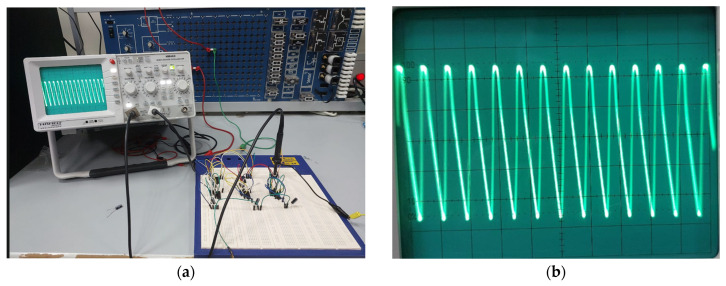
Experimental validation of the proposed memristor-based oscillator: (**a**) Measurement setup using a CD4007 discrete prototype and (**b**) measured output waveform at C=1 μF.

The oscillation principle of the proposed dual memristor-based ring oscillator was experimentally validated using a discrete prototype built around the CD4007UB CMOS integrated circuit, which contains complementary pairs of MOSFETs suitable for emulating the DTMOS configuration by externally connecting the gate and substrate terminals. The complete experimental setup is shown in Figure 8a. Each memristor emulator was constructed using two CD4007 transistors configured as DTMOS devices, with an external state capacitor *C* = 1 μF and discrete PMOS pull-up transistors forming the three-stage ring oscillator core. The output waveform was captured using a digital oscilloscope. It is important to emphasize that this discrete prototype serves a dual-purpose: first, to provide qualitative validation of the oscillation principle, and second, to investigate whether the proposed DTMOS memristor emulator could function correctly in a practical circuit implementation under real-world conditions. Despite its minimalist two-transistor topology, the emulator must demonstrate robust operability within a complete oscillator system, overcoming non-idealities such as component mismatches, parasitic elements, and measurement probe loading that are often absent in idealized simulations. The successful generation of stable oscillation under these practical challenges provides critical evidence that the memristive feedback mechanism is not merely a simulation concept but a practically realizable circuit element. The prototype successfully exhibited stable oscillation, with a measured fundamental frequency of 330 kHz for *C* = 1 μF. The substantial frequency difference between this measurement and the multi-GHz simulated results arises from fundamental differences between the discrete and integrated implementations. The CD4007 transistors are fabricated in a legacy ~5 µm technology, featuring effective channel lengths much longer than the 0.18 µm devices used in our simulated design. Furthermore, the discrete prototype employs a 1 μF external state capacitor, larger than the integrated MOS varactors, which proportionally increases the memristor time constant. Combined with substantial parasitic elements from bond pads, PCB traces, package inductance, and oscilloscope probe loading, factors that are absent or minimized in the integrated implementation, these factors collectively account for the observed higher-frequency scaling between the prototype and the simulated CMOS design.

As depicted in Figure 8b, the output waveform deviates significantly from an ideal sinusoidal shape, exhibiting a non-smooth, asymmetrical profile. This behavior is entirely consistent with expectations for a non-optimized discrete implementation and can be attributed to several factors. First, the CD4007 transistors are designed for general-purpose digital switching at 5–15 V supplies, not for analog subthreshold or high-speed operation, resulting in abrupt transitions and nonlinear current–voltage characteristics that introduce high-frequency harmonics. Second, the large discrete capacitor (1 μF) creates a millisecond-scale time constant that forces slow memristor state evolution, yet the CD4007 switching transitions remain sharp, producing a waveform that resembles a distorted, slew-rate-limited square wave rather than a pure sinusoid. Third, the prototype lacks the fine capacitive tuning, balanced complementary DTMOS matching, and parasitic optimization available in the integrated CMOS version, exacerbating non-idealities such as parasitic capacitances, bond-wire inductances, and mismatched threshold voltages between discrete devices. Additionally, the loading effect of the oscilloscope probe further degrades the waveform quality at the measurement node. Despite these imperfections, the prototype successfully achieves its intended validation objectives. Critically, it confirms that the memristive feedback mechanism operates correctly in practice, the oscillator starts reliably without external triggering, and that it maintains stable oscillation over extended measurement periods. We have separately validated the memristor emulator’s pinched hysteresis behavior using the same CD4007 prototype as depicted in Figure 1c, confirming that the core memristive element functions as intended at the device level. The successful system-level oscillation demonstrated in Figure 8b, despite the considerable limitations of the discrete implementation, provides strong evidence that the memristor-based oscillator topology is sound and translates correctly to integrated form.

Furthermore, this experimental validation demonstrates that the DTMOS memristor emulator is not merely a theoretical construct but a practically realizable circuit element capable of operating within complex systems using readily available, off-the-shelf components. The fact that oscillation was achieved with no specialized fabrication, no post-processing, and no trimming underscores the robustness and accessibility of the proposed design approach. This qualitative validation, combined with the comprehensive simulations in 0.18 µm CMOS presented throughout Section 4, provides confidence that the proposed architecture achieves GHz-range operation with ultra-low power consumption when properly integrated. The quantitative performance metrics—multi-GHz frequencies, sub-100 µW power consumption, clean sinusoidal waveforms, and low phase noise—remain achievable only in full-custom CMOS integration, where careful layout, device matching, parasitic minimization, and optimized DTMOS implementation are possible. The discrete prototype thus serves as an essential bridge between simulation and practical realization, confirming both the theoretical soundness and the practical operability of the proposed memristor-based oscillator architecture.

### 4.2. Chirp Frequency Mode

Beyond fixed-frequency oscillation, the proposed dual-mode oscillator can be reconfigured to operate as a programmable chirp pulse generator, a critical function for frequency-modulated biomedical applications such as neural stimulation, bio-impedance spectroscopy, swept-frequency radar-based vital sign monitoring and wireless biomedical signal processing. In this mode, the memristor’s state capacitor C is increased to a sufficiently large value such that the state voltage $v_{C}(t)$ can no longer track the instantaneous oscillation; instead, it integrates the AC signal over many cycles, producing a slow, monotonic ramp in memristance. This gradual change in $R_{mem}(t)$ continuously modulates the stage delay, resulting in a linear frequency sweep (chirp) at the oscillator output. Unlike conventional voltage-controlled oscillators that require external ramp generators or digital frequency synthesizers, the proposed architecture generates the chirp autonomously using only the memristor’s inherent state dynamics. This section presents comprehensive simulation results characterizing the chirp generation capability of the oscillator, including the effects of state capacitor value and the width of the control signal *SW_chirp_*, on chirp rate, sweep range, and linearity.

**Figure 9:**
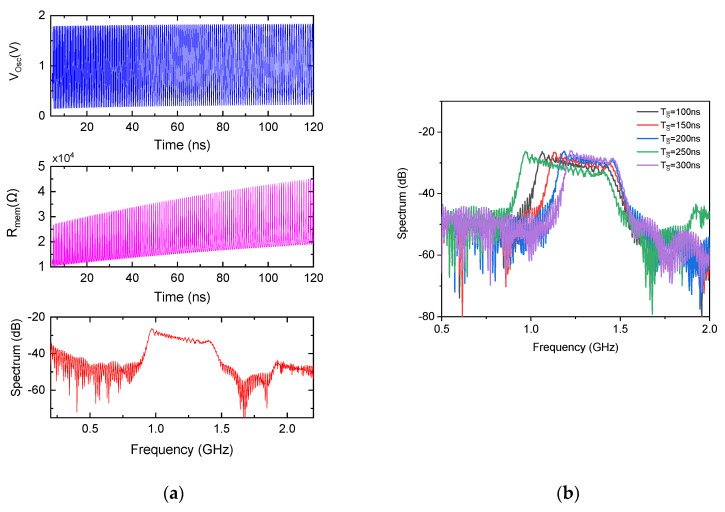
Chirp mode characterization of the proposed dual-mode oscillator: (**a**) Transient output waveform, instantaneous memristance $R_{mem}(t)$ and output spectrum for C=40 pF; (**b**) spectrum dependence on ${SW}_{chirp}$ control signal width.

The chirp mode operation of the proposed dual-mode oscillator was first characterized through transient simulation with a fixed state capacitor C=40 pF, at $V_{DD}=1.8$ V and $W_{var}=1$ μm. Figure 9a presents the transient output waveform, exhibiting a clear frequency sweep over time. The corresponding instantaneous memristance $R_{mem}(t)$, also plotted in Figure 9a, reveals a distinctive dynamic behavior: the memristance oscillates periodically between a minimum and maximum value within each oscillation cycle, but crucially, both the lower and upper bounds of this oscillation increase monotonically with time. This gradual upward drift in memristance is the direct consequence of the state capacitor integrating the AC signal, causing $v_{C}(t)$ to rise slowly over thousands of cycles. As $R_{mem}(t)$ increases, the stage delay grows, and the oscillation frequency progressively decreases, producing a positive-to-negative chirp (downsweep). Figure 9a also depicts the corresponding output spectrum, showing a broad, continuous frequency spread from approximately 1.0 GHz to 1.5 GHz, confirming successful chirp generation.

The output spectrum exhibits a slight positive variation of approximately 4 dB across the chirp bandwidth, with lower-frequency components displaying significantly higher spectral magnitude than higher-frequency components. This phenomenon does not reflect amplitude variation in the time-domain waveform but rather the nonlinear frequency–time relationship inherent to the memristor-based chirp generation mechanism. As the state capacitor integrates charge, the rate of frequency change df/dt is not constant; the sweep progresses rapidly through the upper frequency band and gradually decelerates as memristance increases, causing the oscillator to dwell longer at lower frequencies. Consequently, more oscillation cycles contribute to the low-frequency portion of the spectrum, yielding greater integrated energy and higher FFT magnitude. This behavior is characteristic of passive, integration-based chirp synthesis and confirms that frequency modulation, not amplitude flatness, is the dominant information carrier. For biomedical applications such as swept-impedance spectroscopy or frequency-modulated neural stimulation, this energy concentration at the sweep endpoint is often desirable, as it maximizes signal energy in the band of interest without requiring additional power.

To investigate the programmability of the chirp waveform, the duration of the ${SW}_{chirp}$ control signal, which enables the current injection path to the state capacitor, was varied while maintaining a fixed C=40 pF. Figure 9b presents the output spectra for five different ${SW}_{chirp}$ pulse widths. Notably, the upper bound (highest frequency) of the chirp spectrum remains largely invariant with respect to the control signal width, as the initial memristance state is identical at the moment the chirp is triggered. However, the lower bound (lowest frequency) exhibits strong dependence on ${SW}_{chirp}$ width: longer enable pulses allow the state capacitor to integrate charge over an extended duration, driving $v_{C}(t)$ to higher values and memristance to larger magnitudes, thereby extending the frequency sweep further downward. This behavior provides a simple, digitally controllable approach for adjusting the chirp span without modifying analog components. For instance, increasing the ${SW}_{chirp}$ pulse width from 100 ns to 300 ns expands the sweep range from 1.23 to 1.45 GHz to 0.9–1.42 GHz, a 300 MHz extension of the low-frequency boundary.

**Figure 10:**
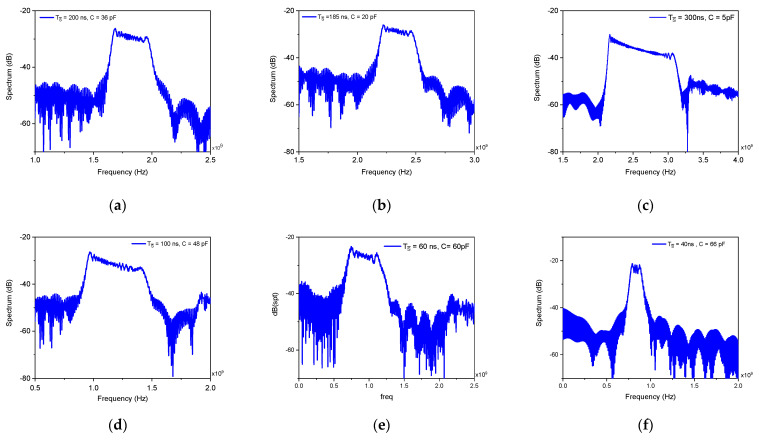
Chirp spectrum dependence on state capacitor C and ${SW}_{chirp}$ pulse width: (**a**) C = 5 pF, (**b**) C = 20 pF, (**c**) 36 pF, (**d**) C = 48 pF, (**e**) C = 60 pF and (**f**) C = 66 pF.

As predicted theoretically, the absolute frequency band of the chirp is primarily governed by the value of the state capacitor C, while the sweep depth is independently controlled by the ${SW}_{chirp}$ pulse width. Figure 10 presents the output spectra for different capacitance values, each measured with a correspondingly adjusted ${SW}_{chirp}$ width to maintain a consistent sweep range. Increasing C shifts the entire chirp spectrum upward: the 5 pF case achieves a maximum frequency of 3.3 GHz, while for 66 pF the spectrum centers around 1 GHz. This positive correlation between capacitance and frequency arises because larger C reduces the quiescent state voltage $v_{C0}$, lowering the initial memristance and thereby raising the oscillation frequency when the chirp is triggered. Simultaneously, for a fixed ${SW}_{chirp}$ pulse width, the sweep depth contracts with increasing C, as the same injected charge produces a smaller voltage increment $\Delta v_{C}=I_{chirp}\cdot T_{SW}/C$. Thus, C serves as the master frequency-setting element in chirp mode, determining the spectral band, while ${SW}_{chirp}$ width independently controls the sweep depth. This orthogonal control, capacitance for coarse band selection, pulse width for fine sweep range adjustment, provides exceptional flexibility for biomedical applications, enabling dynamic reconfiguration of both carrier frequency and modulation bandwidth without additional circuitry or power overhead.

## 5. Discussion and Comparative Analysis

To evaluate the performance of the proposed memristor-based ring oscillator against existing designs, a comprehensive comparison with recently reported CMOS ring voltage-controlled oscillators (VCOs) is presented in Table 5. The comparison focuses on key metrics relevant to biomedical and low-power applications, including oscillator structure, technology node, supply voltage, power consumption, output frequency, phase noise, tuning range, and figure of merit (FoM). The tuning range percentage is calculated using [17,18,19,20,21]:

(8) $$\mathrm{Tuning}\;\mathrm{Range}\;\left(\%\right)=\left(\frac{F_{max}-F_{min}}{F_{avg}}\right)\times 100$$

The figure of merit (FoM), which is the most widely used expression for normalizing phase noise performance against carrier frequency and power consumption in VCOs, is given by [22,23]:

(9) $$\mathrm{FoM}=L\left\{\Delta f\right\}-20log\left(\frac{f_{o}}{\Delta f}\right)+10log\left(\frac{P_{diss}}{1\;\mathrm{mW}}\right)$$

where L{Δf} is the phase noise at offset frequency Δf, $f_{o}$ is the center frequency, and $P_{diss}$ is the power dissipation in milliwatts.

The most striking advantage of the proposed design is its exceptionally low power consumption of 111 µW at 4.017 GHz operation. This represents a reduction of 2.6 to 27 times compared to the reference designs, which consume between 0.285 mW [21] and 3 mW [17]. This improvement is attributed to two key factors. First, the zero-static-power nature of the DTMOS memristor emulator ensures that no DC current is drawn except during switching events. Second, the memristor-based pull-down configuration inherently limits the crowbar current that plagues conventional CMOS inverters during switching transitions. Despite operating in a mature 0.18 µm CMOS process, the proposed oscillator achieves a phase noise of −87.82 dBc/Hz at 1 MHz offset, which is 6.8 dB better than the next-best design [17], which also uses 0.18 µm technology, and significantly outperforms all other references. This exceptional phase noise performance in a ring oscillator topology is particularly noteworthy, as ring oscillators typically exhibit higher phase noise than their LC-tank counterparts. The improvement stems from the inherent filtering action of the memristor dynamics, which smooths the switching transitions and reduces timing jitter. Furthermore, the absence of static current paths eliminates a significant source of low-frequency flicker noise upconversion. The proposed oscillator achieves an extraordinary 133.6% fractional frequency coverage (0.8–4.017 GHz) through its dual-mode operation, the highest tuning range among all compared designs, surpassing even [21] (121.3%) and [19] (96.98%). This wide coverage is not achieved through conventional varactor tuning alone but through the reconfigurable memristor dynamics: fixed-frequency mode provides continuous tuning in the 3.142–4.017 GHz band, while chirp mode extends coverage down to 0.8 GHz through programmable sweeps. Additionally, it is particularly noteworthy that the proposed design achieves its superior performance in a 0.18 µm CMOS process, whereas several comparison designs utilize more advanced 90 nm nodes [18,19,20,21]. Despite the older technology, the memristor-based oscillator delivers better phase noise, lower power, and a wider tuning range than all 90 nm references. This demonstrates that the architectural innovation, integrating a DTMOS memristor into the oscillator core, is more impactful than mere technology scaling for achieving energy-efficient, high-performance frequency generation. The calculated FoM of −169.45 dBc/Hz for the proposed oscillator represents a substantial improvement of 23.5 dB over [21] (−145.91 dBc/Hz) and 26.5 dB over [17] (−143 dBc/Hz). This FoM, which simultaneously accounts for phase noise, frequency, and power consumption, confirms that the memristor-based design achieves an exceptional trade-off among these competing metrics. The combination of low-power, high-frequency, and excellent phase noise positions this oscillator as one of the most energy-efficient ring VCOs reported in the literature, particularly considering the mature 0.18 µm technology node.

The proposed memristor-based dual-mode oscillator offers several distinctive advantages that make it exceptionally well suited for integration into wireless biomedical systems, including implantable medical devices, wearable health monitors, and emerging ultra-wideband (UWB) applications. In the context of wireless biomedical telemetry, the oscillator’s ultra-low power consumption of only 111 µW at 4.017 GHz represents a transformative advancement for energy-constrained implants. For example, in a leadless cardiac pacemaker, where device longevity is critical and battery replacement requires invasive surgery, reducing oscillator power by 2.6 to 27 times compared to conventional CMOS ring VCOs can extend operational lifetime by years or enable battery-less operation when combined with body-heat thermoelectric harvesting. This dramatic improvement stems from the zero-static-power DTMOS memristor, which eliminates DC bias currents entirely and ensures that precious harvested energy is directed primarily to data transmission rather than wasted as heat, a critical consideration for implants where tissue heating must remain below regulatory limits of 1–2 °C. Beyond basic telemetry, the oscillator’s exceptional frequency coverage of 133.6% (0.8–4.017 GHz) through dual-mode operation addresses the growing need for multi-standard interoperability in modern medical devices. A single implantable sensor node today may need to communicate via the MICS band (402–405 MHz) for deep-tissue uplink, the ISM band (915 MHz) for body-area network communication, and Bluetooth Low Energy (2.4 GHz) for smartphone data relay to clinicians. Conventional designs require separate oscillators or complex phase-locked loops for each band, consuming prohibitive area and power. The proposed oscillator, with its continuous coverage from 0.8 GHz to 4.017 GHz, can serve all these functions from a single compact core, dramatically reducing silicon footprint and system complexity. For a multi-channel neural recording implant, this translates directly to smaller device size, a critical factor for cortical applications where millimeter-scale form factors determine surgical feasibility and tissue trauma.

The proposed memristor-based oscillator serves as a versatile core for ultra-wideband (UWB) biomedical transmitters [24,25], generating both impulse-radio and frequency-modulated UWB pulses directly from its programmable chirp mode without requiring additional pulse-shaping or modulation circuitry. This capability enables high-data-rate applications such as wireless capsule endoscopy with real-time video transmission through body tissue, multi-channel neural recording from hundreds of electrodes, and non-contact vital sign monitoring via frequency-modulated continuous-wave radar, all while consuming only 111 µW. With wide frequency coverage spanning 0.8–4.017 GHz and excellent phase noise of −87.82 dBc/Hz, a single oscillator can operate across multiple UWB regulatory bands, including the medical imaging spectrum of 3.1–4.8 GHz, while maintaining spectral purity essential for coexistence with hospital equipment. The ultra-low power consumption approaches levels required for battery-less operation from energy harvesters, enabling permanent cardiac implants that stream ECG continuously without replacement surgery. By integrating pulse generation, frequency synthesis, and modulation into one compact, zero-static-power core, the oscillator paves the way for a new generation of autonomous medical devices: ingestible cameras, high-resolution neural interfaces, and wearable fall-detection systems that communicate wirelessly with unprecedented energy efficiency and regulatory compliance.

The oscillator’s exceptional phase noise of −87.82 dBc/Hz at 1 MHz offset, representing a 6.8 dB improvement over comparable designs, directly impacts clinical reliability in challenging wireless environments. Consider a deep-brain stimulation implant communicating through several centimeters of attenuating brain tissue and skull: lower phase noise translates to lower bit-error rates for a given transmit power, ensuring that critical therapeutic parameters are received correctly despite the hostile propagation environment. Alternatively, for a given error rate, the improved phase noise allows higher-order modulation schemes, increasing data throughput for high-resolution neural recordings, from 64-channel to 256-channel arrays, without expanding bandwidth or increasing power. The figure of merit of −169.45 dBc/Hz confirms that this phase noise performance is achieved without sacrificing power efficiency, a critical balance for thermally safe implants where total power dissipation must remain below 10–20 mW to avoid tissue damage.

Finally, the oscillator’s compact topology and digital programmability make it readily integrable into complex biomedical system-on-chip architectures. It can be placed adjacent to low-noise amplifiers for biosignal acquisition, analog-to-digital converters for digitization, and baseband processors for data compression, all on the same die without thermal crosstalk or interference. The memristor’s zero-static-power nature ensures no thermal coupling with sensitive analog front ends, while its control via simple digital signals ($V_{tune}$ and ${SW}_{chirp}$) allows dynamic reconfiguration under microcontroller supervision. For a closed-loop epilepsy management system, this means the same oscillator can generate carrier frequencies for data uplink during normal monitoring, switch to chirped waveforms for therapeutic stimulation when seizure activity is detected, and recalibrate its frequency in real time to compensate for tissue growth or electrode degradation, all autonomously without external intervention. By demonstrating that a single compact circuit can replace multiple conventional oscillators while consuming less power and offering new functionality, this work paves the way for truly versatile, long-lasting, and intelligent implantable and wearable medical devices capable of sensing, processing, and communicating across multiple bands with unprecedented energy efficiency.

## 6. Conclusions

This paper has presented a novel dual-mode memristor-based ring oscillator designed for energy-efficient wireless biomedical signal conditioning. By integrating a compact DTMOS memristor emulator into a three-stage PMOS ring oscillator core, the proposed architecture achieves two distinct operating modes within a single topology: fixed-frequency mode (3.142–4.017 GHz), consuming only 111 µW, and programmable chirp mode starting from 0.8 GHz for frequency-modulated biomedical applications. The oscillator demonstrates exceptional performance in 0.18 µm CMOS, including phase noise of −87.82 dBc/Hz at 1 MHz offset, a 133.6% overall frequency coverage, and a figure of merit of −169.45 dBc/Hz. Comprehensive simulations confirm robust operation across process corners and temperature variations, while experimental validation using a discrete prototype verifies the oscillation principle. With its ultra-low power consumption, wide frequency coverage, excellent phase noise, and dual-mode reconfigurability, the proposed oscillator is ideally positioned for next-generation wireless biomedical systems, including implantable telemetry, neural stimulation, ultra-wideband transmitters, and non-contact vital sign monitoring. By demonstrating that a single compact circuit can replace multiple conventional oscillators while consuming less power and offering enhanced functionality, this work establishes the memristor-based dual-mode oscillator as a foundational building block for implantable and wearable medical devices.

## Figures and Tables

**Table 1:** Width of the proposed dual oscillator for L = 0.18 μm.

Transistors	Width (μm)
M1, M2, M3	5
Mp1, Mp2, Mp3	0.5
Mn1, Mn2, Mn3	1

**Table 2:** Frequency variation in the proposed dual oscillator at different values of *V_dd_* and *V_sub_*.

*V_tune_* (V)	Frequency (GHz)								
	*V_dd_*				*V_sub_*				
	1.7 V	1.8 V	1.9 V	2 V	1 V	1.2 V	1.4 V	1.6 V	1.8 V
0.4	3.267	4.017	4.781	5.756	4.867	4.750	4.583	4.350	4.017
0.6	3.242	3.975	4.75	5.737	4.842	4.725	4.558	4.317	3.975
0.8	3.2	3.925	4.706	5.706	4.808	4.683	4.517	4.267	3.925
1	3.125	3.858	4.644	5.662	4.775	4.642	4.458	4.2	3.858
1.2	3.008	3.742	4.563	5.606	4.717	4.575	4.383	4.108	3.742
1.4	2.8	3.550	4.438	5.513	4.625	4.475	4.258	3.958	3.550
1.6	2.567	3.300	4.201	5.394	4.508	4.333	4.100	3.767	3.300
1.8	2.342	3.142	4.025	5.175	4.392	4.208	3.958	3.608	3.142

**Table 3:** Simulation results of the three-stage dual-ring oscillator with varying *V_tune_* for different MOS varactor widths.

*V_tune_* (V)	$W_{\mathrm{var}}\;=\;1\;\mu$m		$W_{\mathrm{var}}\;=\;2\;\mu$m		$W_{\mathrm{var}}\;=\;3\;\mu$m	
	Freq. (GHz)	<*R_mem_*> (10^3^ Ω)	Freq. (GHz)	<*R_mem_*> (10^3^ Ω)	Freq. (GHz)	<*R_mem_*> (10^3^ Ω)
0.4	4.017	3.6	3.5	3.015	3.158	2.424
0.6	3.975	3.555	3.442	2.921	3.125	2.352
0.8	3.925	3.485	3.358	2.798	3.075	2.257
1	3.858	3.405	3.267	2.636	3.025	2.142
1.2	3.742	3.311	3.158	2.449	2.958	2.049
1.4	3.550	3.12	3.042	2.193	2.892	1.875
1.6	3.300	2.884	2.933	1.953	2.833	1.736
1.8	3.142	2.526	2.867	1.716	2.808	1.532

**Table 4:** Simulation results of the five-stage dual-ring oscillator with varying *V_tune_* for different MOS varactor widths.

*V_tune_* (V)	$W_{\mathrm{var}}\;=\;1\;\mu$m		$W_{\mathrm{var}}\;=\;2\;\mu$m		$W_{\mathrm{var}}\;=\;3\;\mu$m	
	Freq. (GHz)	<*R_mem_*> (10^3^ Ω)	Freq. (GHz)	<*R_mem_*> (10^3^ Ω)	Freq. (GHz)	<*R_mem_*> (10^3^ Ω)
0.4	1.475	55.2845	1.225	36.702	1.075	23.2325
0.6	1.458	54.6845	1.192	35.252	1.05	22.225
0.8	1.425	51.9085	1.158	32.9875	1.025	20.5975
1	1.392	48.752	1.117	29.9465	0.992	18.736
1.2	1.325	44.201	1.067	25.7025	0.958	16.134
1.4	1.233	37.867	1	21.091	0.917	13.8085
1.6	1.125	30.8935	0.933	17.612	0.883	12.4815
1.8	1.058	25.952	0.908	16.07	0.865	11.4075

**Table 5:** Comparison of performance with existing ring VCO designs.

Parameter	[17]	[18]	[19]	[20]	[21]	This Work
VCO Structure	CMOS RO	CMOS RO	CMOS RO	CMOS RO	CMOS RO	CMOS-Memristor-based RO
No. of delay stages	3	3	5	3	3	3
Technology (µm)	180	90	90	90	90	180
Supply voltage (V)	1.8	1.1	1.8	1.2	1	1.8
Power consumption (mW)	3	1.89	0.289	0.290	0.285	0.111
Output frequency (GHz)	2.96	12.89	5.1	2	6.227	4.017
Phase Noise @ 1 MHz (dBc/Hz)	−79	−43.38	−73.08	−81	−64.57	−87.82
Tuning Range (%)	51.91	-	96.98	80	121.3	133.6
FoM	−143	−122.82	-	-	−145.91	−169.45

## Data Availability

Data are contained within the article.
